# Genetic exploration of Dravet syndrome: two case report

**DOI:** 10.1186/s13256-024-04514-2

**Published:** 2024-04-23

**Authors:** Agung Triono, Elisabeth Siti Herini, Gunadi

**Affiliations:** 1 Department of Child Health, Faculty of Medicine, Public Health and Nursing, Universitas Gadjah Mada, Dr. Sardjito Hospital, Jl. Kesehatan No. 1, Yogyakarta, 55281 Indonesia; 2 Pediatric Surgery Division, Department of Surgery/Genetics Working Group/Translational Research Unit, Faculty of Medicine, Public Health and Nursing, Universitas Gadjah Mada, Dr. Sardjito Hospital, Yogyakarta, 55281 Indonesia

**Keywords:** Dravet syndrome, *SCN1A*, Genetic, Case series, Next-generation sequencing

## Abstract

**Background:**

Dravet syndrome is an infantile-onset developmental and epileptic encephalopathy (DEE) characterized by drug resistance, intractable seizures, and developmental comorbidities. This article focuses on manifestations in two Indonesian children with Javanese ethnicity who experienced Dravet syndrome with an *SCN1A* gene mutation, presenting genetic analysis findings using next-generation sequencing.

**Case presentation:**

We present a case series involving two Indonesian children with Javanese ethnicity whom had their first febrile seizure at the age of 3 months, triggered after immunization. Both patients had global developmental delay and intractable seizures. We observed distinct genetic findings in both our cases. The first patient revealed heterozygous deletion mutation in three genes (*TTC21B*, *SCN1A*, and *SCN9A*). In our second patient, previously unreported mutation was discovered at canonical splice site upstream of exon 24 of the *SCN1A* gene. Our patient’s outcomes improved after therapeutic evaluation based on mutation findings When comparing clinical manifestations in our first and second patients, we found that the more severe the genetic mutation discovered, the more severe the patient’s clinical manifestations.

**Conclusion:**

These findings emphasize the importance of comprehensive genetic testing beyond *SCN1A*, providing valuable insights for personalized management and tailored therapeutic interventions in patients with Dravet syndrome. Our study underscores the potential of next-generation sequencing in advancing genotype–phenotype correlations and enhancing diagnostic precision for effective disease management.

## Background

Dravet syndrome (DS), previously known as severe myoclonic epilepsy of infancy (SMEI), is an infantile-onset developmental and epileptic encephalopathy (DEE) characterized by drug resistance, intractable seizures, and comorbidities including intellectual disability, behavioral problems, sleep disturbances, gait disturbances, and an increased risk of sudden unexpected death in epilepsy [1, 2]. The incidence of DS is approximately 1 in every 15,700 births [3]. The first symptom of DS is seizures in the first year of life, followed by developmental delay [1]. This first seizure is either generalized tonic–clonic or focal (occasionally hemiclonic) clonic, and in more than half of the cases, it is a febrile seizure, making it difficult to distinguish from a self-limiting febrile seizure. Infection, hot environment, exhaust, sunlight, or exercise can initiate an attack of DS [4, 5]. Approximately 80% of patients with DS carry a pathogenic variant of the sodium channel alpha 1 subunit (*SCN1A*) gene resulting in haploinsufficiency Nav1.1, the alpha-1 subunit of the sodium channel. *PCDH19, SCN2A, SCN8A, SCN1B, GABRA1, GABRB3, GABRG2, KCNA2, CHD2, CPLX1, HCN1A,* and *STXBP1* variants may also be involved in DS or DS-like phenotypes. Accordingly, genetic testing is required to identify other genes that play a role in the DS phenotype and to expand genotype-DS phenotype correlations to enhance the future management of this disease [6]. In the last decade, next-generation sequencing (NGS) technology has been able to analyze a set of genes (targeted panel sequencing), exome [(whole exome sequencing (WES)], or genome [whole genome sequencing (WGS)] in a single sequencing process, making it possible to diagnose rare diseases such as early childhood epilepsy [7]. Identification of the genetic basis of DS can provide additional information regarding pathophysiology, prognosis, and individual drug therapy options according to the patient’s condition.

We present a case series involving two children, one aged 11 years and 2 months, and the other aged 1 year and 4 months. Both children were diagnosed with DS, exhibiting symptoms of intractable seizures, global developmental delay, and seizures triggered by postimmunization fever. Despite displaying similar symptoms, the two individuals possess different genetic variants of the *SCN1A* gene and also possible novel mutation in DS. We also discuss the main clinical characteristics, treatment course, and management of DS at tertiary referral hospitals in Indonesia.

## Case presentation

### Patient 1

**Fig. 1:**
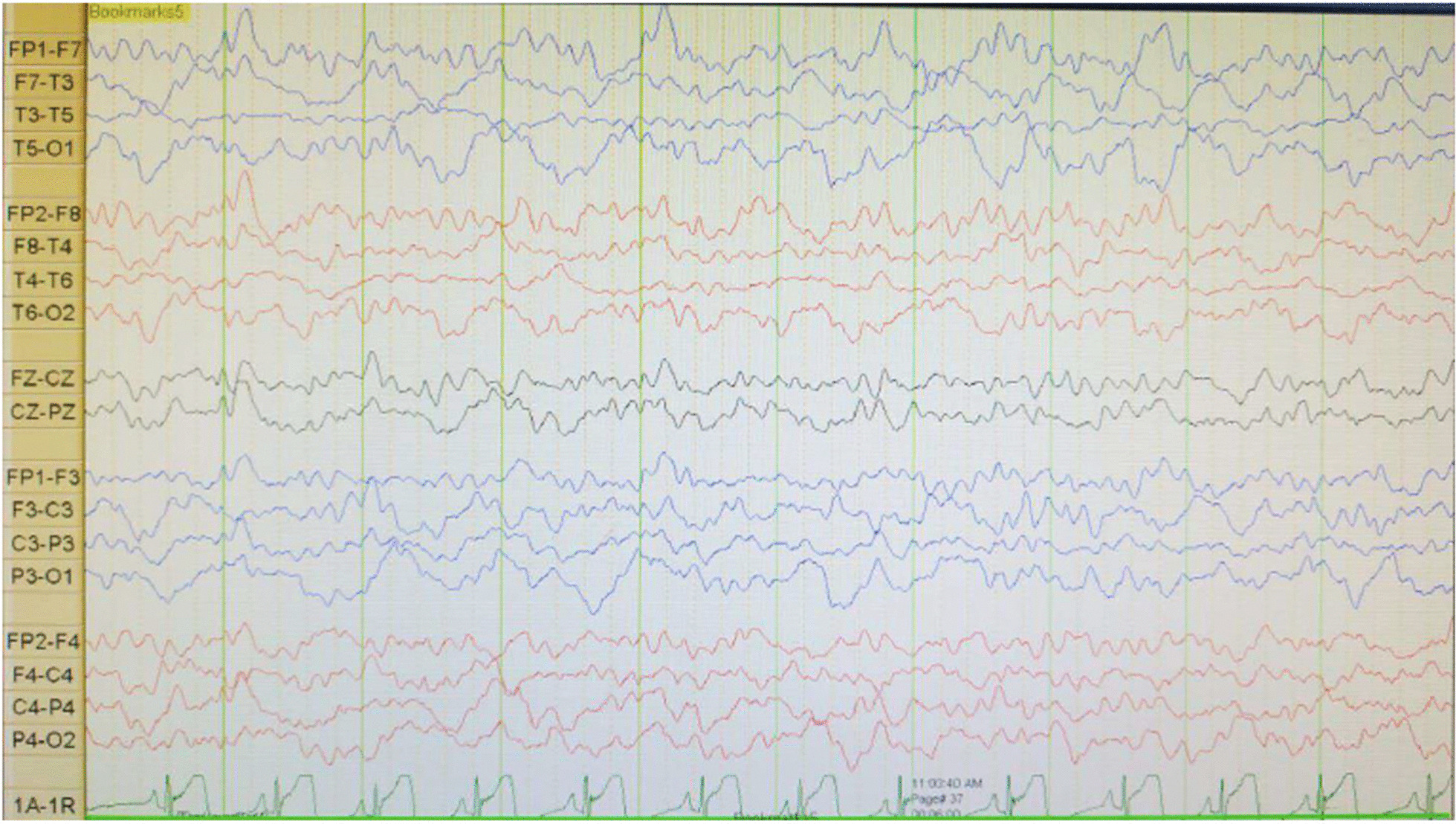
Electroencephalography (EEG) shows diffuse epileptiform irritative abnormality on a normal background

**Fig. 2:**
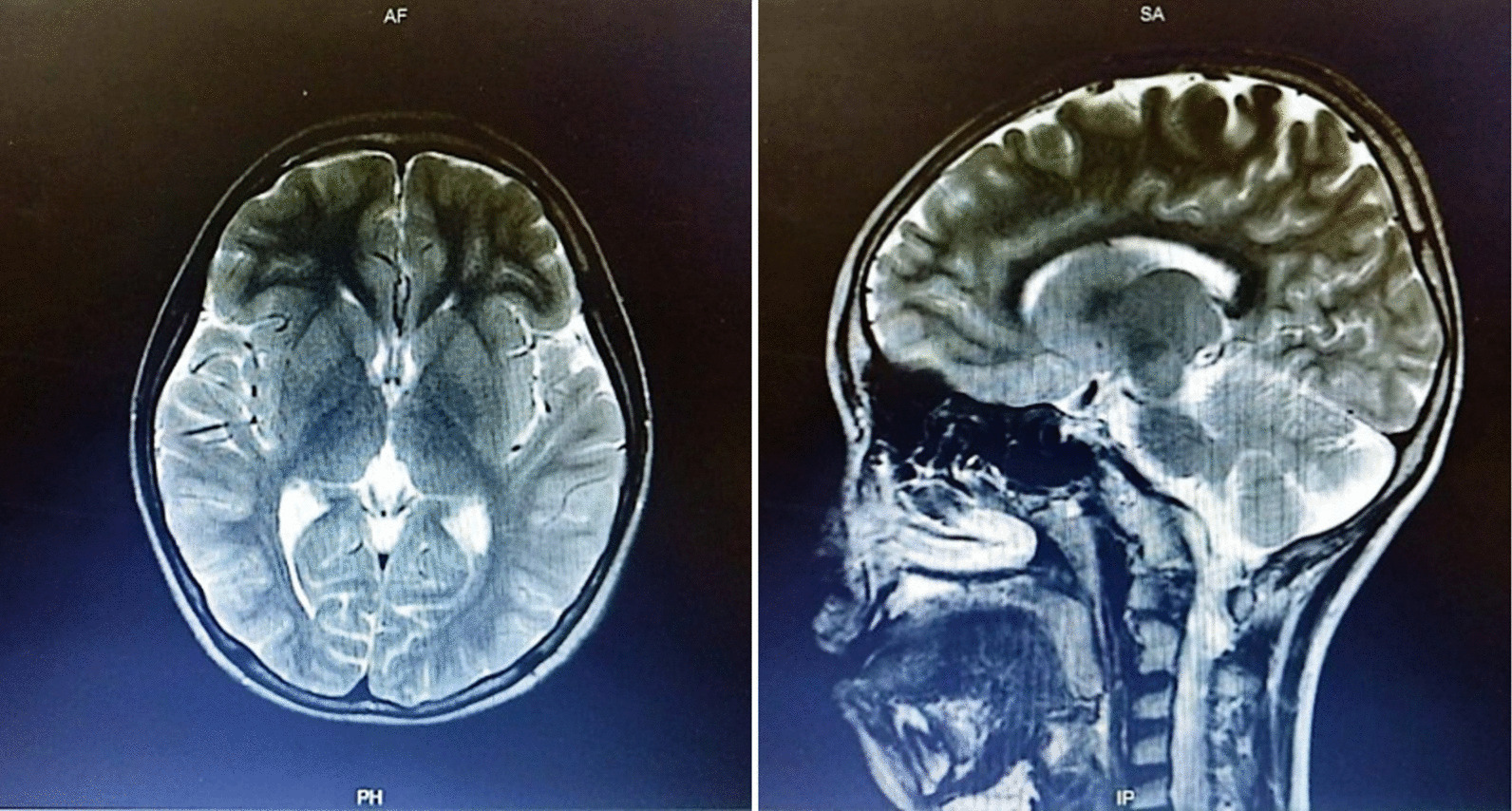
Axial brain magnetic resonance imagery shows cerebral atrophy, bilateral frontal subarachnoid enlargement, bilateral occipital lobe polymicrogyria, and a neuroglial cyst in the right temporal lobe

A boy with Javanese ethnicity aged 11 years and 2 months with uncontrollable seizures regularly visits our hospital. The patient had his first seizure at the age of 3 months with a duration of 15 min, and it was triggered after receiving diphtheria–pertussis–tetanus (DPT) immunization, which was accompanied by fever. The patient has about six to seven seizures per day for 1 min in the form of generalized tonic–clonic and absence seizures. He was the first child of nonconsanguineous healthy parents with normal prenatal and birth history. He has a younger sister with normal development. There is no history of family members with febrile seizure. The patient was born at 40 weeks of gestation, with a birth weight of 4000 g, length of 52 cm, and head circumference of 33 cm. The patient is currently experiencing global developmental delay and is still in kindergarten. He had learning difficulties and was unable to speak words at an age-appropriate level. He had delayed motor development and was unable to perform age-appropriate motor activities. Head circumference was 46.5 cm (microcephaly). There were no signs of meningeal irritation nor Babinski response. The motor examination revealed no increased tone in the upper and lower limb. Other systemic examinations revealed no abnormalities. Interictal electroencephalography (EEG) showed diffuse epileptiform irritative abnormality on a normal background (Fig. 1). Magnetic resonance imaging (MRI) of the brain showed cerebral atrophy, bilateral frontal subarachnoid enlargement, bilateral occipital lobe and polymicrogyria, and a neuroglial cyst in the right temporal lobe (Fig. 2). He was recommended to get genetic testing done since he was suspected of having DS.

Whole genome sequencing (WGS), whole exome sequencing (WES), and Sanger sequencing were performed at 3Billion (Seoul, Korea). The WGS and WES procedures were conducted according to the protocols of Richards *et al*. [8] and Seo *et al*. [9], respectively. Both WES and WGS are comprised of four main parts: (1) high-quality sequencing; (2) sequencing data analysis including alignment to the genome reference consortium human 37 (GRCh37)/hg19 for WES, also alignment to the genome reference consortium human 38 (GRCh38) and revised Cambridge reference sequence (rCRS) of the mitochondrial genome for WGS; (3) variant annotation and prioritization by EVIDENCE [a software that was developed in house to prioritize variants based on the American College of Medical Genetics and Genomics (ACMG) guidelines [10]]; and (4) variant interpretation in the context of the patient’s symptoms and reporting of disease-causing variants. Once EVIDENCE prioritizes the top candidate variants/genes, 3Billion’s highly-trained clinical/medical geneticists manually curate each variant to identify the disease-causing variant for reporting.

In our initial examination, we performed WES on patient 1 and subsequently identified a copy number variant (CNV), prompting us to proceed with WGS. The WGS analysis revealed a heterozygous pathogenic 552.9 Kb deletion variant in 2q24.3. The heterozygous deletion NC_000002.12:g.165811316_166364199delinsTGTACACTA at 2q24.3 spans across three genes (*TTC21B*, *SCN1A*, and *SCN9A*). The variant is not observed in the gnomAD SVs v2.1.1 dataset. *SCN1A* is subject to haploinsufficiency. Other pathogenic variants have been reported in this region. There are multiple similarly affected individuals reported with similar likely pathogenic copy–number–loss overlapping this region [11, 12]. Therefore, this variant was classified as pathogenic. Due to region-spanning mutation in SCN1A, which suitable with clinical manifestation, the patient was diagnosed with DS (OMIM 607208: since we were unable to perform a Sanger sequencing study on both of the parents, the pattern of inheritance is still uncertain.

The arents were counseled about their child’s condition and agreed to undergo multipronged therapy. Before the patient was diagnosed with DS, he had received valproic acid (30 mg/kg per day), phenobarbital (2.5 mg/kg per day), and oxcarbazepine (5 mg/kg per day), also physio, occupation, and speech therapy but had not shown significant improvement. He was seizure-free for 3 months after oxcarbazepine was changed to levetiracetam (27 mg/kg per day). However, the patient then had another episodes of less than 5 minutes general tonic–clonic seizure (GTCS)-induced by fever. Interictal EEG was performed to evaluate his condition, and we found that the diffuse epileptiform irritative abnormality persisted.

### Patient 2

A 1 year and 4 month-old-girl with Javanese ethnicity was referred to our hospital due experiencing myoclonic seizure followed by 20 minute GTCS at 3 months, after fever following DPT immunization. She then continued to experience generalized tonic–clonic seizures one to two times per day for 10–15 seconds. At 9 months of age, the patient received a second DPT immunization, and on the same day, she had another generalized tonic–clonic seizure that lasted > 30 minutes, resulting in her admission to the pediatric intensive care unit. Before the first seizure, the patient could lift her head, grasp a toy and make eye contact, but after that, she could neither lift her head nor grasp an object. The patient has no previous history of trauma.

**Fig. 3:**
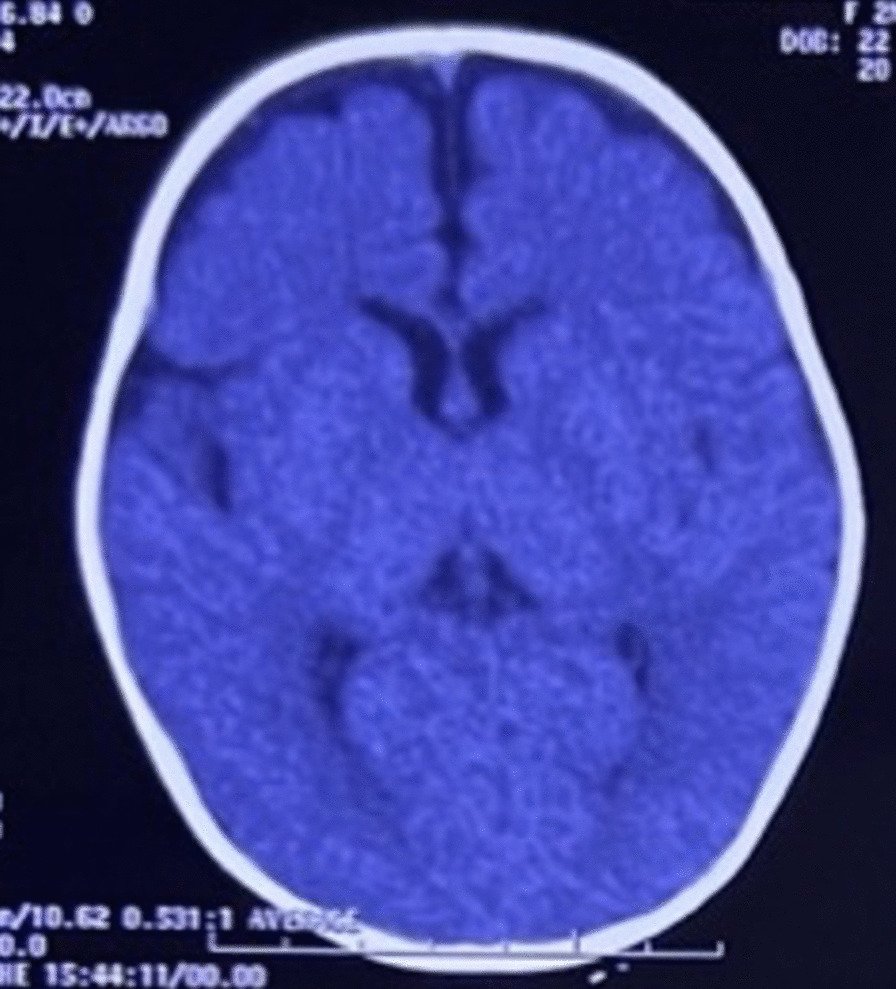
Axial brain computed tomography scan shows a subdural hygroma in the right and left frontoparietal region, without any other abnormalities

**Fig. 4:**
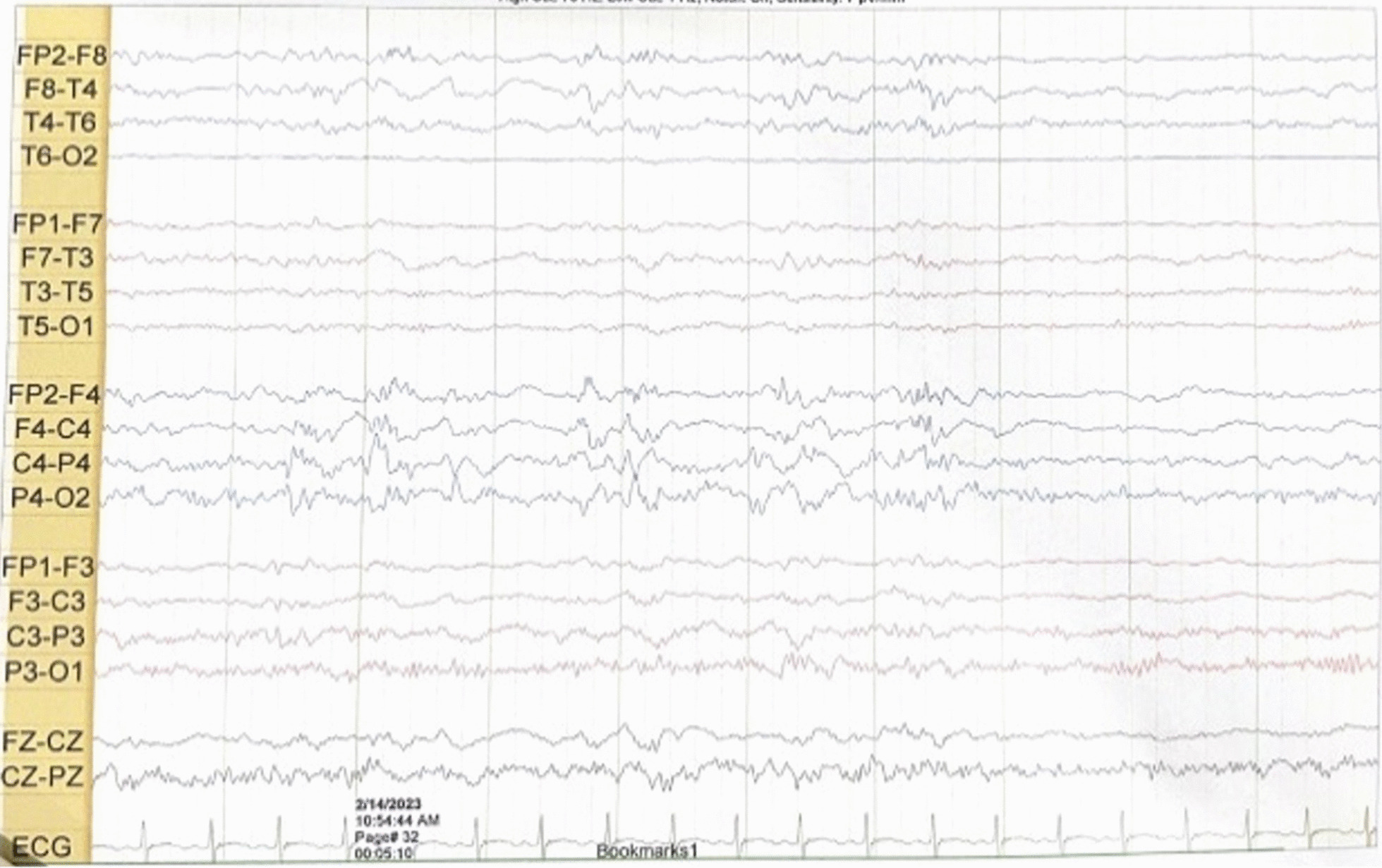
Electroencephalography shows abnormal irritative epileptiform with a normal background

She had a normal head circumference increased physiological reflexes in all extremities. Other systemic examinations revealed no abnormalities. Computed tomography (CT) scan examination of the head showed a subdural hygroma in the right and left frontoparietal region, without any other abnormalities (Fig. 3). Electroencephalography (EEG) at the beginning of the seizure did not show any abnormalities, but the EEG follow-up 7 months after the onset of the seizure showed an abnormal epileptiform (spike wave) with a normal background (Fig. 4). Thus, she was suspected of having DS and was recommended to undergo genetic examination.

**Fig. 5:**
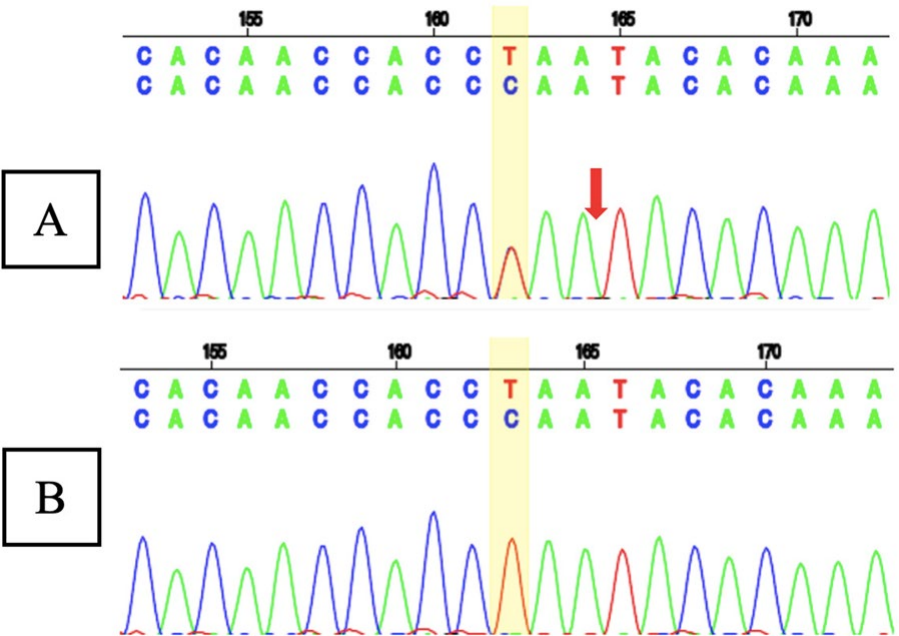
**A** Sanger sequencing result of patient 2 showed a heterozygous mutation of the *SCN1A* gene with the genomic position 2-166859265-T-C (GRCh37), [NM_001165963.4:C.4003-2A >G [NP_001159435.1:p.?] (red arrow); and **B** Sanger sequencing result of patient 2’s mother showed normal sequence

Whole exome sequencing (WES) showed a likely pathogenic variant identified as a heterozygous mutation of the *SCN1A* gene with genomic position 2-166859265-T-C (GRCh37), [NM_001165963.4:C.4003-2A > G [NP_001159435.1:p.?]. The variant is located in the canonical splice site upstream of exon 24 of *SCN1A* gene (NM_001165963.4 transcript). Since this variant is an essential splicing variant, the protein consequence is uncertain and therefore represented as (p.?). In this patient’s genetic mutation, the canonical junction site occurs which is expected to alter the junction and result in loss or disruption of normal protein function. However, using an in silico predictor, spliceAI (https://spliceailookup.broadinstitute.org/), the variant is predicted to result in a loss of 22 base pairs at end of exon 24. This loss is expected to create a frameshift at the Gly1342 position. Sanger sequencing confirmed the patient’s genotype (Fig. 5A), but the mother’s Sanger analysis was negative (Fig. 5B). Due to familial issues, Sanger sequencing was not performed on the father, leaving the inheritance pattern unresolved.

The parents were counseled about their child’s condition and agreed to undergo multipronged therapy. Before patient was diagnosed with DS, she received clonazepam (0.01 mg/kg per day), valproic acid (29 mg/kg per day), and phenytoin (5 mg/kg per day), but seizure persisted. When phenytoin was stopped, with valproic acid (30 mg/kg per day) and clonazepam (0.04 mg/kg per day) adjusted, seizures were greatly decreased. Later, patient only experienced one seizure per year. The patient routinely received physio, speech, and occupational therapy.

When comparing the clinical features and outcomes of the two patients (Table 1), we found that our first patient, who had three medications, was still having a generalized seizure induced by fever with duration less than 5 minutes after they had been seizure-free for 3 months (at the age 11 years and 8 months. Our second patient, however, only experienced one seizure annually after receiving two medications (at the age 1 year and 10 months). This difference implies that the clinical state of the first patient was worse than that of the second.

**Table 1:** Comparison table of the clinical characteristics between case 1 and case 2

Characteristics	Case 1	Case 2
Seizure onset	3 months, triggered after immunization	3 months, triggered after immunization
Seizure type	Generalized tonic clonic seizures, absence seizure	Generalized tonic clonic seizures, myoclonic, and status epilepticus
Family history	None	None
Developmental	Global developmental delay	Global developmental delay
Neurologic status	Normal	Increased physiological reflexes in all extremities
Electroencephalography features	Diffuse epileptiform abnormality on a normal background	Abnormal epileptiform with a normal background
Radiology	Head magnetic resonance imaging result: Cerebral atrophy, bilateral frontal subarachnoid enlargement, bilateral occipital lobe polymicrogyria, and neuroglial cyst in right temporal lobe	Head computed tomography scan result: Subdural hygroma in the bilateral frontoparietal region
Genetic testing	Heterozygous deletion NC_000002.12:g.165811316_166364199delinsTGTACACTA at 2q24.3 spans across three genes (TTC21B, SCN1A, and SCN9A)	Heterozygous mutation in exon 9 of the *SCN1A* gene, c.4003-2A > G (p.?)
Inheritance	Unknown	De novo
Outcome	Seizure free for 3 months with valproic acid (40mg/kg per day), phenobarbital (2.5mg/kg per 12 hours), and (levetiracetam 27mg/kg per day), but later, the patient had generalized seizure induced by fever with duration less than 5 minutes. Electroencephalography evaluation showed epileptiform irritative abnormality still persisted	Seizure decreased to one seizure per year, with a less than 1 minute duration after given valproic acid (30 mg/kg weight per day) and clonazepam (0.04 mg/kg body weight per day)

*MRI* Magnetic Resonance Imaging, *EEG* electroencephalography

## Discussion

Research on the identification of DS genetic mutations using NGS has never been done in Indonesia. In 2010, we conducted a study to identify pathogenic variants of the *SCN1A* gene using the Sanger sequencing method and successfully reported cases of novel *SCN1A* mutations in Indonesia in patients with severe myoclonic epilepsy in infancy (SMEI) and borderline SMEI (SMEB). The first boy identified with SMEI experienced a variety of seizures, including his first febrile seizure and general tonic–clonic seizure at 7 months of age, and later suffered from myoclonic seizures, left-sided hemiconvulsions, also focal convulsions without fever, along with delayed speech development. The second patient with SMEB had his first febrile seizures with GTCS after immunization at 3 months old, then later on experienced status epilepticus, GTCS, and atonic convulsions without fever [13]. We also conducted another research on the spectrum of generalized epilepsy with febrile seizure plus (GEFS+) focusing on clinical manifestations and *SCN1A* gene mutations. That study analyzed a total of 34 patients who suffered from SMEI (7 patients), SMEB (7 patients), febrile seizure plus (FS+) and absence/myoclonic/atonic/partial seizures (11 patients), and FS+ (9 patients) [14].

However, the research that we have done uses the Sanger sequencing genetic examination, which is expensive and takes considerable time. Additionally, it is unable to find any other gene besides *SCN1A* in patients with DS. A study by Djémié *et al*. in Belgium reported the discovery of 28 pathogenic variants of the *SCN1A* gene using the NGS method which were previously missed or undiagnosed using Sanger sequencing [7]. To link DS cases more effectively, we are attempting to conduct NGS genetic tests, specifically WES and WGS.

Dravet syndrome (DS) was infrequently reported in Indonesia due to its difficulty in diagnosis, misdiagnosis as febrile seizures or other epilepsy syndromes, or lack of follow-up and genetic testing in our country. According to the to the International League Against Epilepsy (ILAE) [15], the diagnostic criteria for this condition should consist of a number of the following symptoms: (1) a family history of epilepsy or febrile seizures; (2) normal development before seizures onset; (3) seizure before 1 year of age; (4) EEG with generalized spike and polyspike waves; (5) pleomorphic epilepsy (myoclonic, focal, clonic, absence, and generalized seizures); (6) focal abnormalities or early photosensitivity; (7) psychomotor retardation after 24 months; (8) exacerbation of seizures with increased body temperature; and (9) the appearance of subsequent ataxia, pyramidal signs or interictal myoclonus after the beginning of psychomotor slowing. Both of our patients had seizures beginning with increased body temperature and regression of development after seizure onset, which were resistant to the majority of anticonvulsant medications. The seizures began as generalized tonic–clonic seizures, followed by absence seizures. Both of our patients also experienced subsequent ataxia and pyramidal signs. Thus, they were suspected of having DS and were advised to undergo genetic testing.

Infants with DS have normal physical and psychomotor development at the time of their first seizure, which typically occurs between the ages of 5 and 8 months. In our case series, both of our patients experienced their first seizure at the age of 3 months [16, 17]. In the first year of life, the most common form of seizure is febrile tonic–clonic. Some patients may experience myoclonic and dyscognitive seizures infrequently. Frequently, protracted seizures result in status epilepticus. In the first year of life, seizures are precipitated by fever/illness, immunization, and cleansing [16]. As the infant develops, he or she will experience a variety of seizure types, as well as fever and emotional stress, flashes of light, and overexertion being seizure triggers. The child with DS will develop hypotonia, ataxia, incoordination, and pyramidal signs, dysautonomia events, cognitive impairment, and behavioral disturbances such as attention deficit, hyperactivity, or autistic characteristics [15]. Some of the conditions above are very consistent with what happened to our patients.

The EEG performed during the early phases of the disease is normal. However, as the child grows, generalized spike waves with isolated or brief discharges of fast polyspike waves may be present [15, 18]. In the first case, we found diffuse epileptiform irritative abnormality with a normal background, whereas in the second case, initially it was found normal, then a few months later it became abnormal irritative epileptiform with a normal background.

Genetic testing is developing rapidly and playing a significant role in the specific diagnosis and management of epilepsy [19, 20]. Several genes with pathogenic mutations produce DS or DS-like phenotypes, which inevitably require different drug therapy approaches. Genes that cause DS can be grouped based on how they work: specifically, three sodium channel-related genes (*SCN2A, SCN8A*, and *SCN1B*), one potassium channel-related gene (*KCNA2*), three gamma-aminobutyric acid receptors (*GABAR*) genes (*GABRA2, GABRB3*, and *GABRG2*), a cyclic nucleotide gated cation channel gene (*HCN1*), and other functional genes including *CHD2, CPLX1*, and *STXBP1*. Approximately 80% of patients with DS have a pathogenic variant of the *SCN1A* gene, from which the majority of *SCN1A* variants are de novo, but 10% of people inherit the *SCNA1* mutation from one or both parents [6]. Both of our patients had a mutation in the *SCN1A* gene, which is the most common mutation seen in DS.

Furthermore, *TTC21B* and *SCN9A* mutations were also found in our first patient. A study conducted by Suls *et al*. also reported a four generation Bulgarian family with epilepsy, revealing a heterozygous 400 kb deletion on chromosome 2q24 that included the *SCN1A* and *TTC21B* genes [21]. The patients exhibited variable phenotypes, but all experienced generalized tonic–clonic seizures around the first year of life, with some presenting myoclonic or absence seizures. Febrile seizures occurred in three of the four patients during infancy. Notably, one patient had mild mental retardation, another had psychomotor slowing, and a third had mental retardation from early infancy; all showed reduced seizures on medication. The findings in that study parallel the situation observed in our initial patient case. Meanwhile, a study by Singh *et al*. identified a heterozygous mutation in the *SCN9A* gene in two patients diagnosed with DS [22]. One of these patients also exhibited a mutation in the *SCN1A* gene. The study provided evidence suggesting that the *SCN9A* gene on chromosome 2q24 could potentially serve as a modifier for DS. Among 109 patients with DS, 8% were found to have an *SCN9A* mutation. This included six patients with double heterozygosity for *SCN9A* and *SCN1A* mutations and three patients with only heterozygous *SCN9A* mutations, supporting the notion of a multifactorial inheritance pattern [22]. The previous research confirmed the severity of clinical symptoms in our first patient, whom we identified mutations in the *SCN1A, SCN9A*, and *TTC21B* genes.

In the last decade, there has been a very rapid development of neurogenetic science and diagnostic technology. NGS is the latest method of genetic examination that allows for the discovery of causal mutations, including de novo, novel, and familial mutations related to epilepsy syndromes that have variable phenotypic features [23]. The first generation of DNA sequencing using the Sanger method could only examine one gene at a time and had limitations especially when examining large genomic regions, so the NGS method is more widely used today [7, 23]. A study conducted by Kim *et al*. in Seoul reported an increase in diagnostic yield using WES after targeted panel sequencing with negative results in infantile onset epilepsy by 8%. This result suggests that WES assays increase the opportunity to search for new epilepsy genes and uncover less well-known epileptic phenotypes from known neurological diseases [24]. The WES examination also allows for the discovery of de novo or inherited mutations if the patient and both parents are examined [25].

According to the recommendations of the North American consensus panel, clobazam and valproic acid are the first-line therapies for antiepileptic drugs, followed by stiripentol, topiramate and levetiracetam. Patients with a suboptimal response to clobazam and valproic acid have been advised to consider the ketogenic diet as a second-line treatment [17]. *SCN1A* is a gene that codes for sodium channel channels, so drugs that work as sodium channel blockers, such as lamotrigine, phenytoin, carbamazepine, oxcarbazepine, lacosamide, and rufinamide, are contraindicated in patients with DS because they can increase the frequency of seizures [4]. After the failure of first- and second-line therapy, surgical therapies, such as vagus nerve stimulation (VNS), were moderately agreed upon and should be considered [17]. Besides medication, controlling infections and body temperature variations also showed to decrease the frequency of seizures and severity of the disease [18]. Initially, the first patient received oxcarbazepine and the second patient got phenytoin, which had been contraindicated to patients with DS. Futhermore, after eliminating medications that were contraindicated, both patients’ outcome improved.

In this study, we discovered unique mutations that have never been documented before, particularly in Indonesia, where NGS analysis of DS genetic variants has never been done. However, the limitation of this study, is that the information comes from two cases only. Further research is needed to explore more cases from Indonesia population.

## Conclusion

In summary, our case series utilizing next-generation sequencing (NGS) unveils the intricate genetic landscape of Dravet syndrome (DS) in two Indonesian pediatric cases. By using WGS and WES, we identified distinct mutations in the *SCN1A* gene, as well as contributions from genes, such as *TTC21B* and *SCN9A*. The power of WGS lies in its ability to uncover rare pathogenic variants, including a 552.9 Kb deletion in the 2q24.3 region. These findings emphasize the importance of comprehensive genetic testing beyond *SCN1A*, providing valuable insights for personalized management and tailored therapeutic interventions in patients with DS. Our study underscores the potential of NGS in advancing genotype–phenotype correlations and enhancing diagnostic precision for effective disease management. Furthermore, we found that the clinical condition of the first patient was worse than that experienced by the second patient. This difference suggests that the more severe the genetic mutation detected, the more severe the clinical manifestations of the patient.

## Data Availability

The dataset used and/or analyzed during the current study are available from the corresponding author on reasonable request.

## Abbreviations

ACMG: American College Of Medical Genetics
CNV: Copy number variant
CT: Computed tomography
DS: Dravet syndrome
DEE: Developmental and epileptic encephalopathy
EEG: Electroencephalography
FS +: Febrile seizure plus
GEFS +: Generalized epilepsy with febrile seizure plus
GRCh37: Genome reference consortium human 37
GRCh38: Genome reference consortium human 38
GTCS: General tonic clonic seizure
ILAE: International league against epilepsy
MRI: Magnetic resonance imaging
NGS: Next-generation sequencing
rCRS: Revised Cambridge reference sequence
SCN1A: Sodium channel alpha 1 subunit
SMEB: Severe myoclonic epilepsy of infancy-borderline
SMEI: Severe myoclonic epilepsy in infancy
VNS: Vagus nerve stimulation
WES: Whole-exome sequencing
WGS: Whole-genome sequencing
